# Supplementary material for: Antibodies to coagulase of Staphylococcus aureus crossreact to Efb and reveal different binding of shared fibrinogen binding repeats
Source: Front Immunol. 2023 Sep 27;14:1221108. doi: 10.3389/fimmu.2023.1221108 (PMC10565355; doi:10.3389/fimmu.2023.1221108)
Supplement: Supplementary file 1 [file DataSheet_1.pdf]

Supplementary Material

Antibodies to Coagulase of *Staphylococcus aureus* crossreact to Efb and reveal different binding of shared Fibrinogen binding repeats

Federico Bertoglio[1,2,3,*], Ya-Ping Ko[4], Sheila Thomas[4, §], Liliana Giordano[1], Francesca Romana Scommegna[1,&], Doris Meier[3], Saskia Polten[3], Marlies Becker[3], Srishtee Arora[4], Michael Hust[3], Magnus Höök[4] , Livia Visai[1,5]

[1]Department of Molecular Medicine (DMM), Center for Health Technologies (CHT), Unità di Ricerca (UdR) Consorzio Interuniversitario Nazionale per la Scienza e Tecnologia dei Materiali (INSTM), University of Pavia, Pavia, Italy

[2]School of Advanced Studies IUSS Pavia, Pavia, Italy

[3]Department of Medical Biotechnology, Institute for Biochemistry, Biotechnology and Bioinformatics, Technische Universität Braunschweig, Braunschweig, Germany

[4]Center for Infectious and Inflammatory Diseases, Institute of Biosciences and Technology, Texas A&M University Health Science Center, Houston, TX, United States

[5]Medicina Clinica-Specialistica, UOR5 Laboratorio di Nanotecnologie, Istituti Clinici Scientifici (ICS) Maugeri, Istituti di Ricovero e Cura a Carattere Scientifico (IRCCS), Pavia, Italy

[§] Present address: Department of Microbiology and Immunology, East Carolina University, Greenville, North Carolina, USA

[&]Present address: Department of Oncology and Metabolism, Academic Unit of Molecular Oncology, University of Sheffield, Sheffield, UK

*** Correspondence:**
Federico Bertoglio
f.bertoglio@tu-bs.de

1 Supplementary Figures

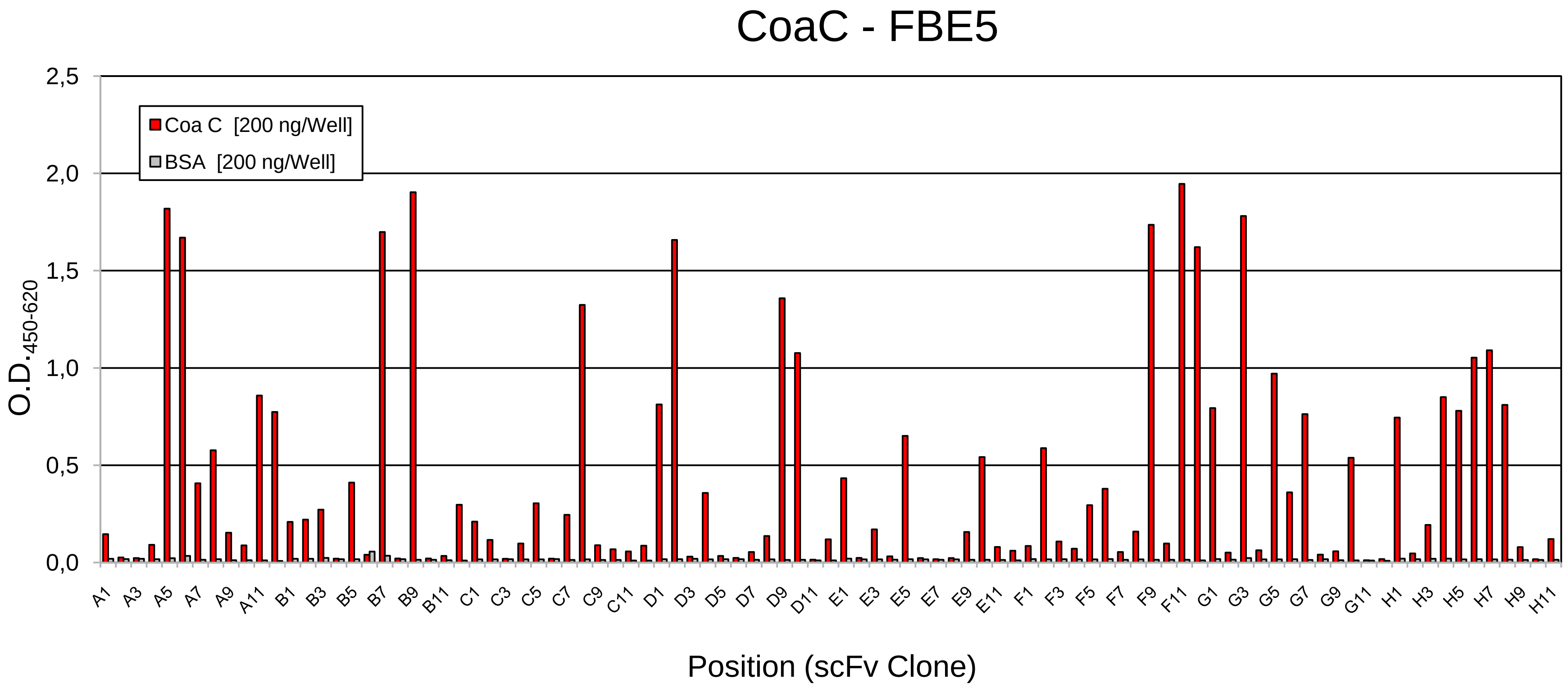

Supplementary Figure 1. Screening ELISA results after 3 panning rounds on CoaC

Both signals (Optical Density 450-620nm) on CoaC and BSA for each of the 95 tested clones are represented as sided bars.

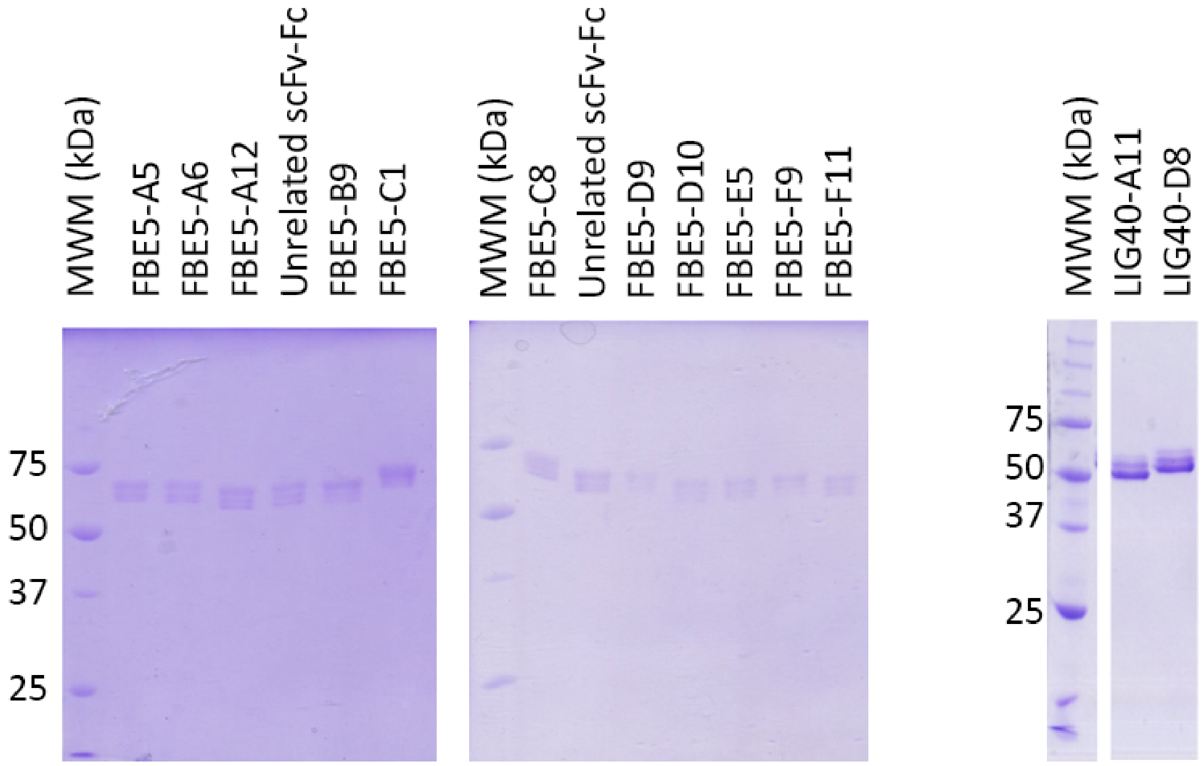

Supplementary Fig. 2- Reducing SDS-PAGE of FBE5 and LIG40 antibodies

SDS-PAGE under reducing conditions of all the scFv-Fc used in this study. Images have been cropped for clarity. MWM (Molecular Weight Marker).

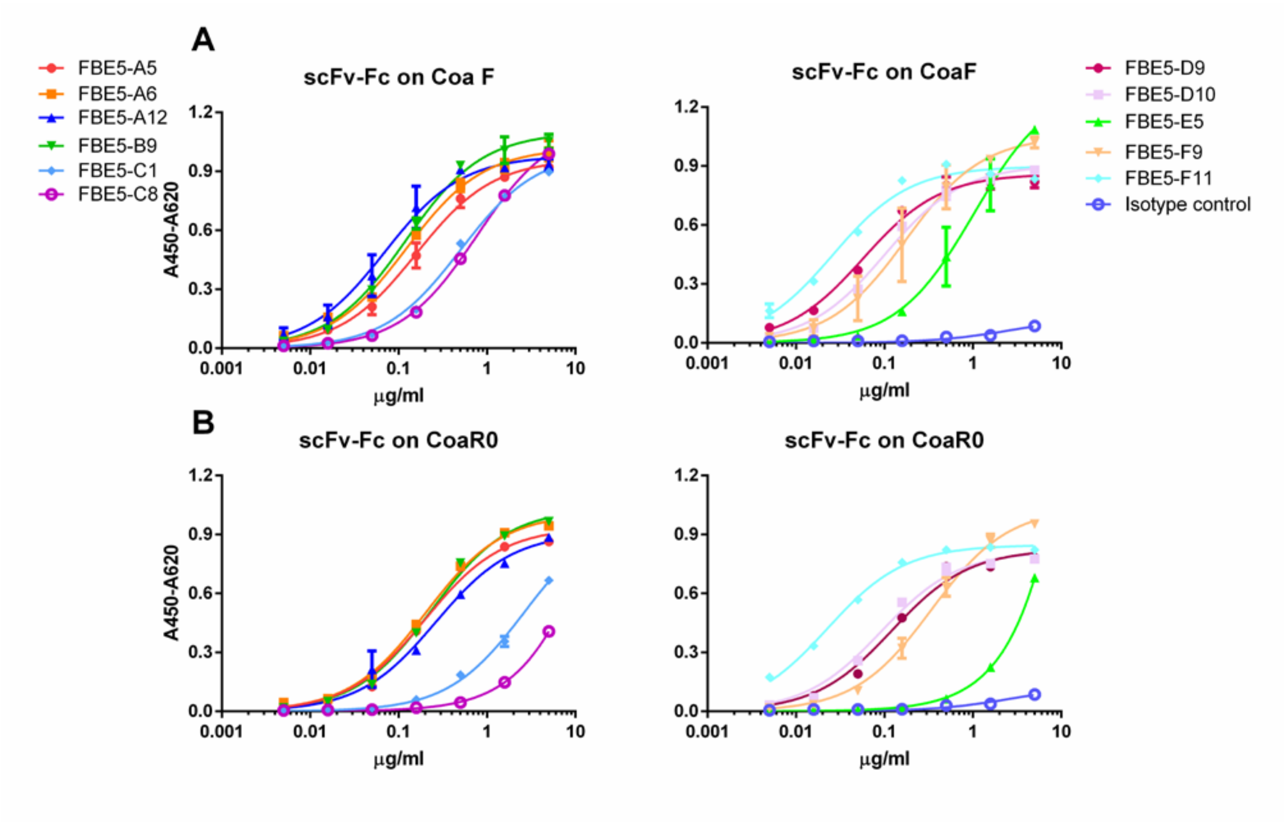

Supplementary Fig. 3- Dose-dependent binding of anti-CoaC mAbs to Coa recombinant proteins

Titration ELISA to investigate binding of FBE5 mAbs to CoaF (A) and CoaR0 (B) recombinant constructs and determine EC50. BSA (not represented) and an unrelated human scFv-Fc were used as negative and isotype controls, respectively.

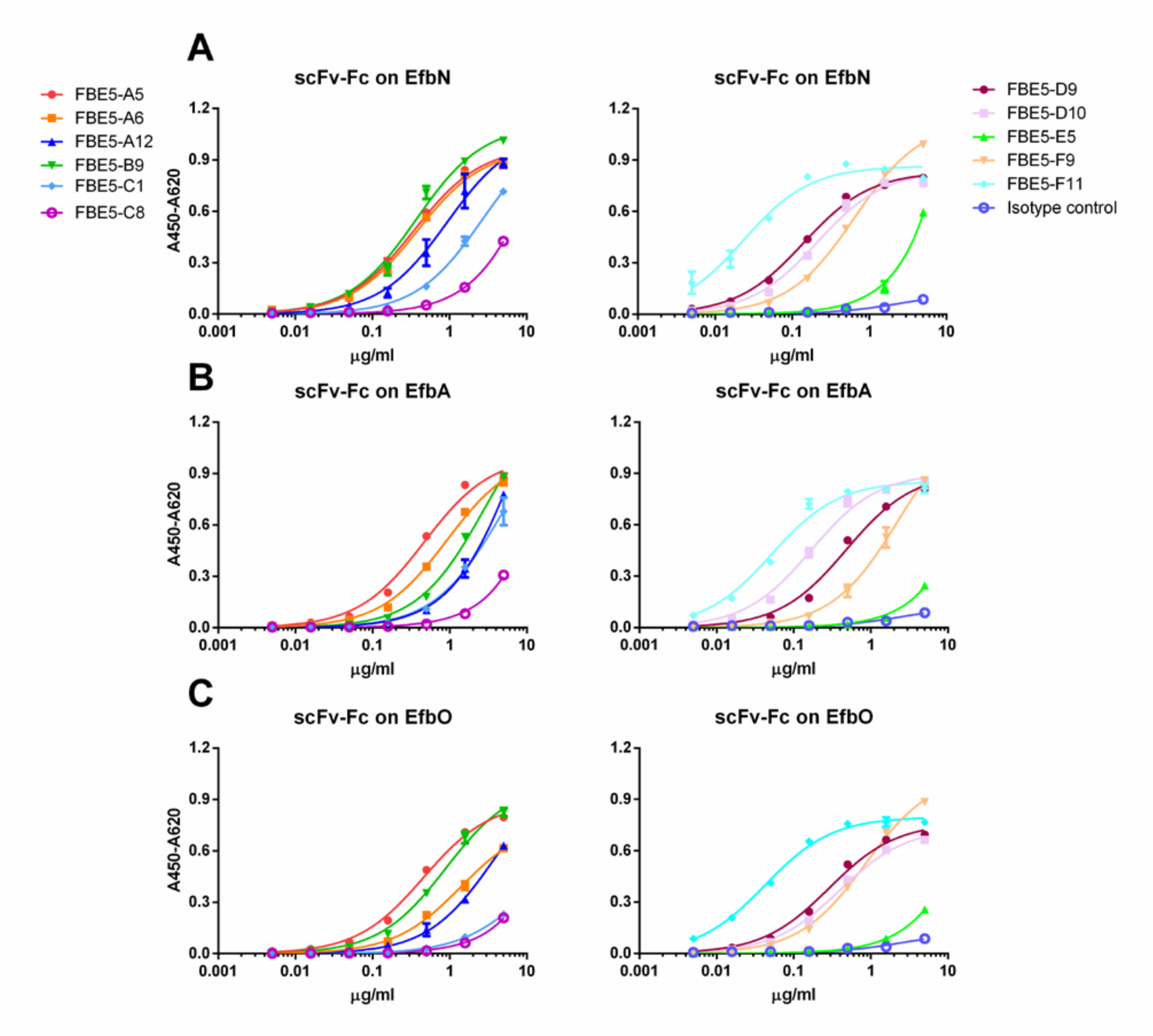

Supplementary Fig. 4 Dose-dependent binding of anti-CoaC mAbs to Efb recombinant proteins

Titration ELISA to investigate binding of FBE5 mAbs EfbN (A), EfbA (B), EfbO (C) recombinant constructs and determine EC50. BSA (not represented) and an unrelated human scFv-Fc were used as negative and isotype controls, respectively.

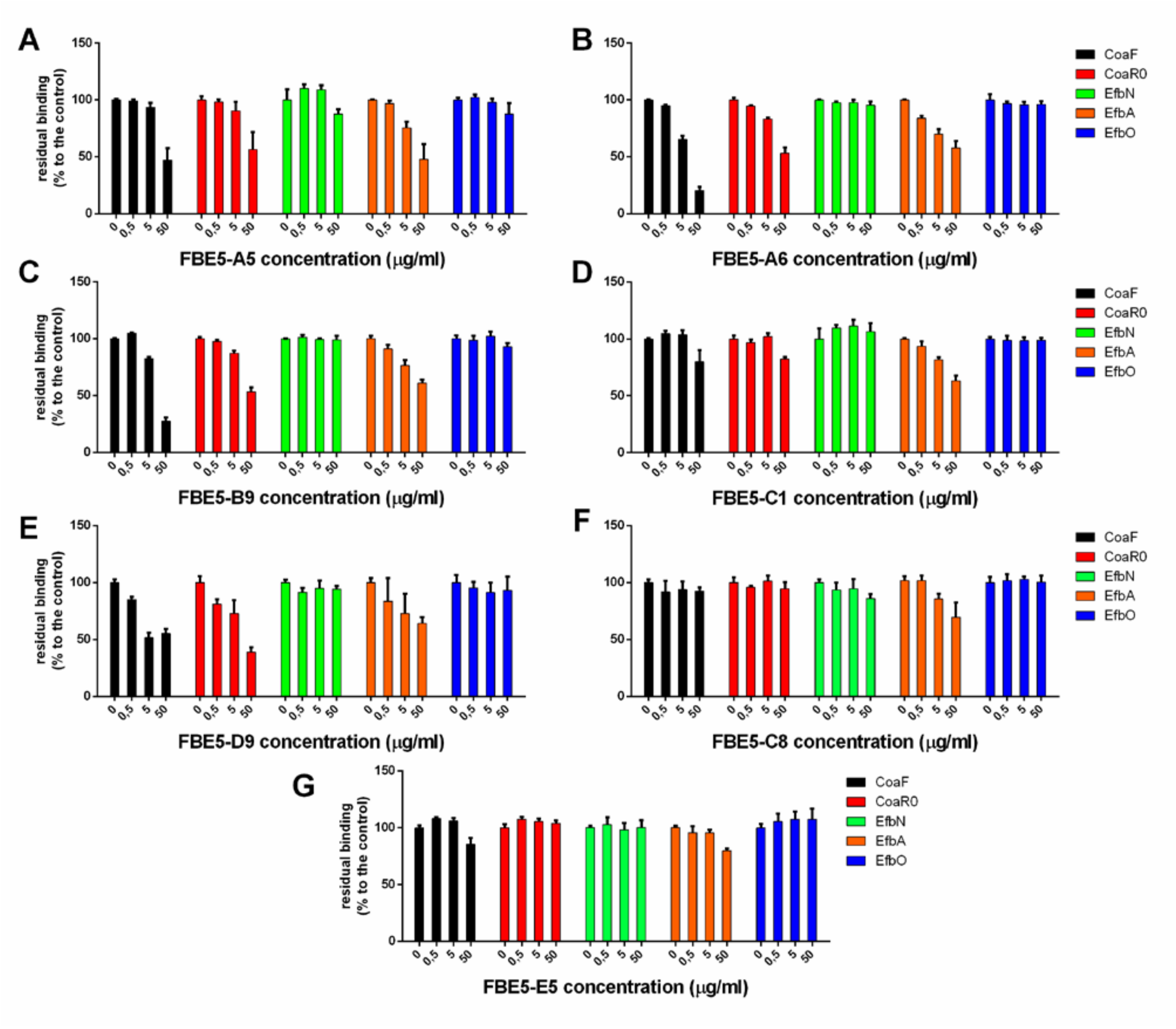

Supplementary Fig. 5 - Anti-CoaC antibodies not inhibiting both Coa and Efb fibrinogen binding activity

Antibodies FBE5-A5 (A), FBE5-A6 (B), FBE5-B9 (C) and FBE5-C1 (D), FBE5-D9 (E), FBE5-C8 (F), FBE5-E5 (G) were pre-incubated at the indicated amounts with the fixed amounts of Coa or Efb recombinant constructs (CoaF, CoaR0, EfbN and EfbO at final concentration of 10nM, EfbA at 750µM) and then transferred on a Fg-coated ELISA plate. The remaining Fg-bound antigens were detected through their tags (GST, except for EfbN that harbours a 6xHis tag). Control wells in which no antibody was added were set to 100% and the residual binding of Coa and Efb constructs were determined by comparing control wells with the ones where indicated amounts of mAbs were added. Average +- SEM of two independent experiments is represented.

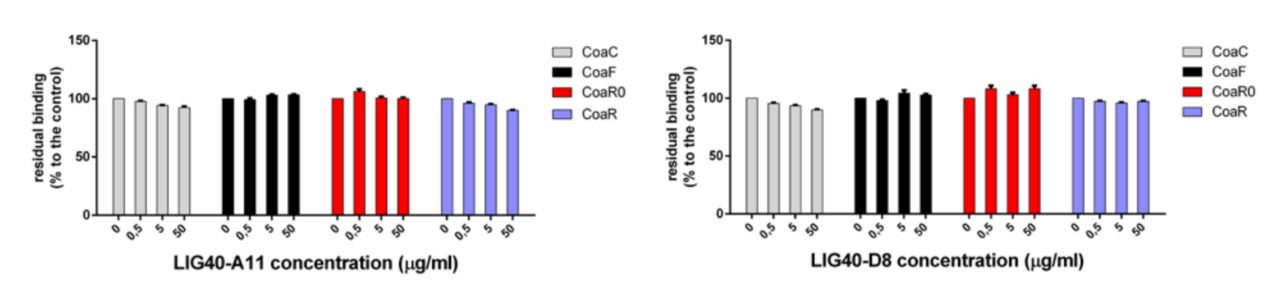

Supplementary Fig. 6 - Anti-CoaR antibodies do not inhibit Coa Fg binding

Antibodies LIG40-A11 (left) and LIG40-D8 (right) were pre-incubated at the indicated amounts with the fixed amounts of Coa recombinant constructs (CoaF, CoaR0, CoaR at final concentration of 10nM, CoaC at 2nM) and then transferred on a Fg-coated ELISA plate. The remaining Fg-bound antigens were detected through their GST tag. Control wells in which no antibody was added were set to 100% and the residual binding of Coa constructs were determined by comparing control wells with the ones where indicated amounts of mAbs were added. Average +- SEM of two indipendent experiments is represented.
